# Can the use of the CROS system provide head shadow effect contribution to unilateral Cochlear Implant Users?

**DOI:** 10.1590/2317-1782/20212021071

**Published:** 2022-04-01

**Authors:** Ana Cristina Hiromi Hoshino, Maria Valéria Schmidt Goffi-Gomez, Paola Angelica Samuel Sierra, Smita Agrawal, Carina Rodriguez, Ana Claudia Martinho de Carvalho, Robinson Koji Tsuji

**Affiliations:** 1 Grupo de Implante Coclear, Departamento de Otorrinolaringologia, Hospital das Clínicas – HC, Faculdade de Medicina – FM, Universidade de São Paulo – USP, São Paulo (SP), Brasil.; 2 Advanced Bionics, Valencia (CA), USA.

**Keywords:** Unilateral Cochlear Implant, Contralateral Routing of Signal, Speech Clarity, Ease of Listening, Head-shadow, Implante Coclear Unilateral, Roteamento de Sinal Contralateral, Clareza da Fala, Facilidade de Escuta, Efeito Sombra da Cabeça

## Abstract

**Purpose:**

The aim of this study was to evaluate the contribution of the CROS system on the head shadow effect in unilateral implant users.

**Methods:**

Prospective cross-sectional study, approved by the ethics committee under protocol 2.128.869. Eleven adults with post-lingual deafness users of unilateral Advanced Bionics CI were selected. Speech recognition was evaluated with recorded words presented at 65dBA at 0^o^ azimuth and at 90^o^ on the side contralateral to the CI, with noise at 55dBA, using CI alone and CI + CROS system. The results were analyzed using paired t-test with a 0.05 alpha.

**Results:**

The mean speech recognition scores were significantly better with CI + CROS in relation to the condition of CI alone (p <0.05, p <0.005 and p <0.005 respectively). In the presentation at 0^o^ azimuth, no significant differences were found.

**Conclusion:**

Users of unilateral CI without useful residual hearing for the use of hearing aids or unable to undergo bilateral surgery can benefit from the CROS device for speech recognition, especially when the speech is presented on the side contralateral to the CI.

## RESUMO

**Objetivo:**

O objetivo deste estudo foi avaliar o efeito do sistema CROS em fenômenos como efeito sombra da cabeça em usuários de implante coclear unilateral.

**Método:**

Estudo transversal prospectivo, aprovado pelo conselho de ética sob protocolo 2.128.869. Onze adultos com surdez de instalação pós-lingual usuários de IC Advanced Bionics unilateral foram selecionados. O reconhecimento de fala foi avaliado com palavras gravadas apresentadas a 65dBA a 0^o^ azimute e a (90^o^ no lado contralateral ao IC), com ruído a 55dBA, usando somente o IC e IC+sistema CROS. Os resultados foram analisados usando teste t pareado com alfa de 0,05.

**Resultados:**

Os escores médios de reconhecimento de fala foram significativamente melhores com IC + CROS em relação à condição apenas IC (p <0,05, p <0,005 e p <0,005 respectivamente). Na apresentação à frente não foram encontradas diferenças significantes.

**Conclusão:**

Os usuários de IC unilateral sem resíduo útil para uso de prótese auditiva ou impossibilitados de submeter-se à cirurgia bilateral podem se beneficiar do dispositivo CROS para o reconhecimento de fala, sobretudo quando a fala for apresentada ao lado contralateral ao IC.

## INTRODUCTION

Although bilateral cochlear implantation is the gold standard in cases of bilateral profound hearing loss^(1,2)^, this treatment is not always achievable due economic or medical reasons. As compared to bilateral CI recipients, unilateral adult as well as pediatric CI recipients demonstrate limited localization of the sound sources, greater difficulty in understanding speech in spatially separated noise, increased listening effort and lower quality of life^(3-5)^.

Listening with two ears allows the CI recipient to overcome head-shadow, a physical phenomenon that results from the head acting as an 'acoustic barrier' to the sounds and noise coming from different positions in space. With unilateral hearing, the CI ear is at a higher SNR when it is ipsilateral to a sound source than when it is contralateral to the sound source. The head-shadow effect results in a 6.4dB attenuation in signal at the contralateral ear and can reach up to 20dB for high frequency speech sounds^(6)^. This may not seem significant but increasing the SNR in this proportion can result in improved speech intelligibility in some environments by as much as 50%^(7)^.

The CROS (Contralateral routing of signals) system is an intervention designed to minimize the short-comings of monaural or asymmetric hearing by transmitting the sounds at the poorer ear to the better ear. By doing so, the listener can gain unattenuated access to sounds contralateral to the better hearing ear, thereby soothing the 'head-shadow' effect^(8-11)^. Recently, the CROS device, which consists of a non-implantable behind-the-ear device has been developed for patients with CIs^(12-17)^. This CROS device, called the Naída Link CROS, transmits a broadband audio signal (8 kHz) at a rate of 300 kbits/sec with minimal delay (2 msec) and minimal power consumption (2 mW) using HiBAN wireless technology. The device also provides access to monaural adaptive as well as binaural beamforming technology^(17)^ for improved face-to-face conversation in noise. Hence, the Naída Link CROS has the potential to bring some aspects of benefits of bilateral implantation to patients who can only obtain a unilateral CI and do not have aidable hearing in the contralateral ear.

The objective of this study was to evaluate whether a CROS device may improve unilateral CI recipient’s speech intelligibility in noise. We hypothesized that use of CROS will improve speech understanding, speech clarity and ease of listening in the head-shadow condition while not causing a detriment in the condition that the sound and noise comes from the front.

## METHODS

This prospective cross-sectional study was approved by the ethical board of the Institution under protocol number 2.128.869. Written informed consent was obtained from all subjects before any tests or procedures were performed.

Eleven post-lingually deaf adults, implanted with Advanced Bionics CIs (CII or later implant) which offers the possibility of CROS use with the contralateral CI speech processor were recruited from the CI patient pool of a tertiary hospital. Eligible participants met the following criteria: at least 6 months of implant use, open set sentence recognition scores of ≥ 60% in quiet and unaided audiometric thresholds ≥80 dBHL at 500 to 2000 Hz in the non-implanted ear. Subjects had no prior experience with CROS at the onset of the study and used the device only during the evaluation.

Table 1 displays demographic information regarding age, etiology of deafness, time of implant use and audiological profile collected from the subject´s clinic files.

**Table 1:** Demographic distribution of the participants

**Demographic variable**	**Parameter(s)**	**Data**
**Sex**	Female	6
	Male	5
**Age (in years)**	Median	47
	Min – max	29 - 55
**Etiology (N)**	Trauma	1
	Policondritis (autoimmune)	1
	Ototoxicity	1
	Unknown	8
**Time of CI use (months)**	Mean (SE)	35.36 (9.38)
	Min – Max	6 – 113
**Contralateral PTA (dBHL)** *	Median	99.55 (4.39)
	Min – Max	80 – 120
**Speech recognition (%)**	Median	91.82 (2.96)
	Min – Max	70 – 100

* unaided condition

**Caption:** SE = standard error; CI = cochlear implant; PTA = pure tone average

**Figure 1:**
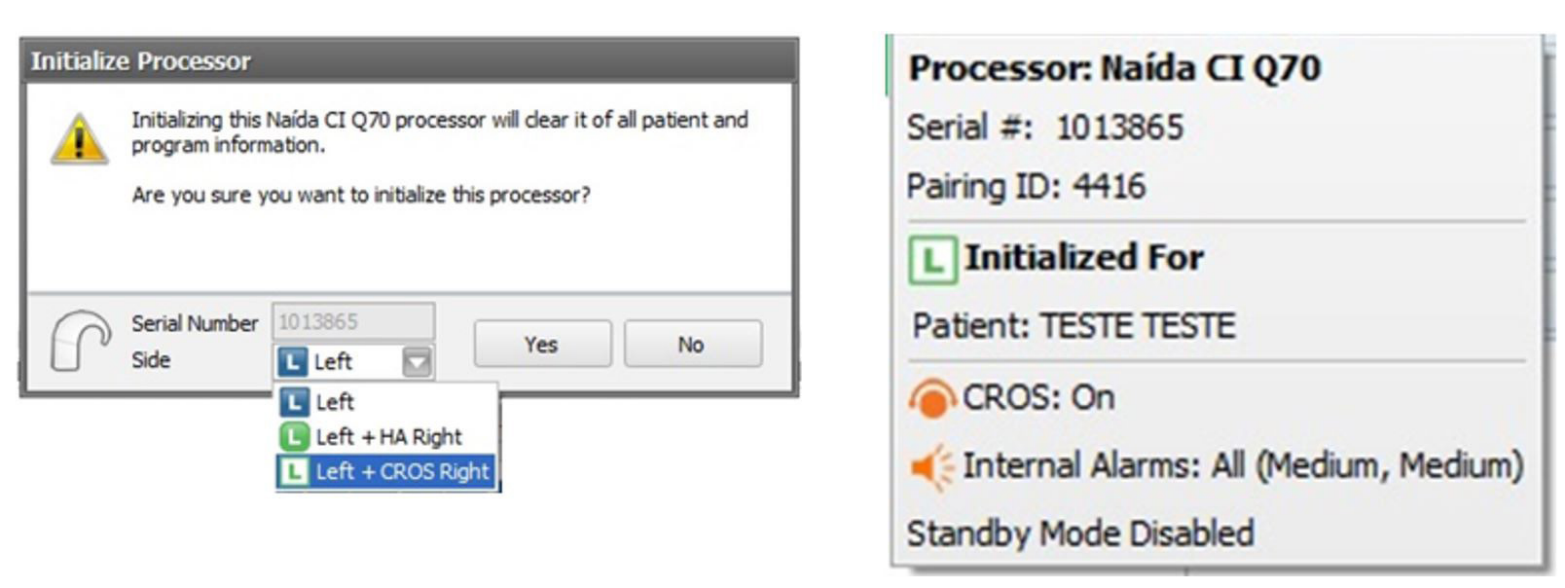
Screen shots of the initialization of the processor to pair the CROS device and confirmation in the downloading task bar

The present study was conducted with Naída CI Q70 sound processor and Naída Link CROS. Participants received a Naída CI Q70 speech processor on loan during the evaluation period of the study. For this, the speech processor was first initialized to function in combination with a CROS system in the contralateral ear. Everyday omnidirectional program was downloaded to the loan processor and CI+CROS initialization was reconfirmed during the downloading process (Figure 1). In the present study, benefit of beamforming was not assessed. Hence, only one omnidirectional program was created and used. It is important to note that no additional programming on the CROS side or balancing of signals between the two devices is needed. CI and CROS signals are automatically mixed in a 50/50 ratio. Also, an adjustment is automatically applied to the CI’s T-Mic input when speech is presented from front in the CI+CROS configuration^(2)^.

Speech recognition was tested with 25-dissyllabic word lists^(18)^ presented at 65dBHL with multi-talker babble noise at 55dBHL, in device conditions of CI speech processor only (CI only) and CI speech processor + CROS (CI+CROS). Speech recognition test were conducted in a clinical double walled sound booth. For head-shadow test condition, speech was presented on the contralateral side of the CI and noise in the ipsilateral side (S_CROS_N_CI_) and for S_0_N_0_ condition, speech and noise were presented at same time in the loudspeaker in front of the patient (0^o^ azimuth).

**Figure 2:**
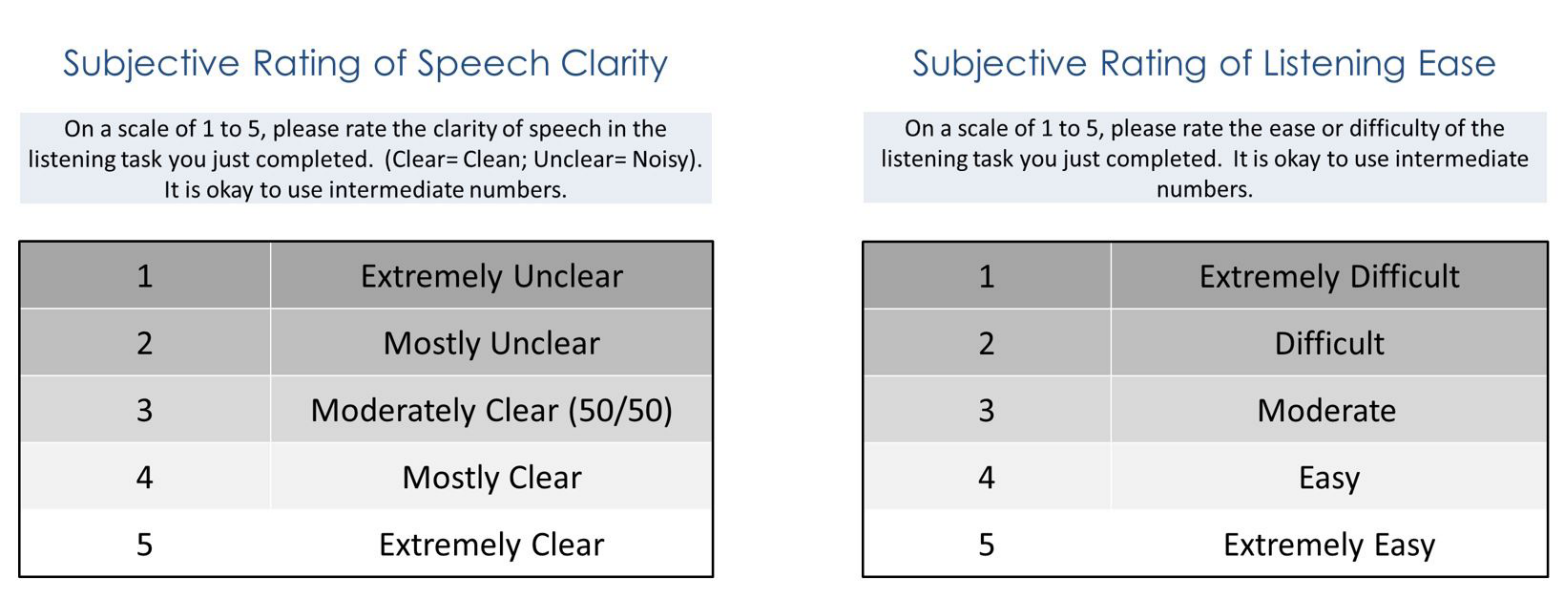
Visual analog scales for speech clarity and ease of listening (or difficulty)

The order of test conditions (CI only and CI + CROS; S_CROS_N_CI_ and S_0_N_0_) was randomized^(19)^. After each test condition was completed, the participants rated the clarity of speech and ease of listening on a 5-point visual analog scale (Figure 2) while still seated in the sound booth. A continuous scale was used where listeners were allowed to use intermediate numbers.

Speech recognition results and subjective ratings across the test conditions were analysed within-subject using paired sample t-tests with an alpha of 0.05.

## RESULTS

Table 2 lists the speech recognition scores, speech clarity and ease of listening ratings under the various test conditions. In the S_0_N_0_ test condition, outcomes with and without CROS were not statistically different. However, in the S_CROS_N_CI_ condition, speech recognition scores were significantly higher in the CI+CROS device condition, (p = 0.0134). The ratings for ease of listening and clarity of speech were also found to be significantly higher with the CI+CROS device condition (p=0.0016 and p=0.0033 respectively).

**Table 2:** Speech recognition (%) scores, clarity of speech and ease of listening ratings without and with the CROS device in the studied test conditions.

	**CI only**		**CI + CROS**		**p-value**
	Mean (SE)	Min - max	Mean (SE)	Min - max	
**S_0_N_0_**					
**Speech recognition scores (%)**	53.45 (5.07)	20 - 80	53.09 (5.03)	24 - 76	0.9520
**Clarity of speech ratings**	3 (0.27)	2 – 5	2.91(0.29)	2 – 5	0.7560
**Ease of listening ratings**	2.81 (0.23)	2 – 4	2.73 (0.27)	1 – 4	0.7560
**S_CROS_N_CI_**					
**Speech recognition scores (%)**	53.82 (4.33)	32 – 76	66.18 (4.59)	36 – 88	0.0134*
**Clarity of speech ratings**	2.64 (0.28)	1 – 4	3.73 (0.28)	3 – 5	0.0033*
**Ease of listening ratings**	2.36 (0.24)	1 – 4	3.36 (0.15)	3 – 4	0.0016*

* statistical significance according to paired sample t-Test comparison

**Caption:** S_0_N_0_ =Speech and noise presented at the front (at 0o azimuth); S_CROS_N_CI._ = Speech presented at the CROS side and noise presented at the side of the cochlear implant (CI); SE = standard error

## DISCUSSION

The objective of the present study was to evaluate the effect of CROS device for improving speech understanding noise that were previously inaccessible to unilateral CI recipients. While improvement in outcomes was observed under head-shadow condition, we did not see evidence for access to S_0_N_0_ condition with a CROS device in our study. In fact, since the CROS system does not involve the auditory pathways of both sides, it is not possible to expect binaural abilities. As head shadow effect depends on the physical barrier of head, the benefit could be revealed.

During the period of the study, a number of studies have been published on benefits of CROS use in CI recipients. The improvement of speech understanding in noise evidenced in our data is consistent with the findings of other groups who have worked with Naída Link CROS^(12-17)^. This finding is also aligned with studies of CROS use with a contralateral hearing aid or normal hearing ear (single-sided deafness) or with wired prototypes for CIs^(20-27)^.

Snapp et al.^(10)^, Dorman et al.^(12)^ and Dwyer et al.^(13)^ included test conditions that evoked summation effect and did find a significant improvement in the CI+CROS condition on a magnitude consistent with that in bilateral listeners. For the reader wondering why CROS use would improve speech understanding from front, that would imply a duplication of the information achieving the same auditory pathway, this is due to the automatic enhancement applied by the CI+CROS system when speech is presented from front^(14)^. We hypothesize that we did not see this effect in our study due to the use of disyllabic words in noise which have lower context and can be a more difficult task. The studies cited above used sentences in noise.

While there is a growing body of work already out there with CI+CROS, the present study, contributes to the literature, since it assessed the performance with portuguese disyllables in noise, while previous studies have assessed CROS benefit with sentences in noise. Kurien et al.^(20)^ included a test condition with monosyllables but in quiet. A second important contribution of our work is demonstrating that CROS benefit can be measured in simple clinical settings even in the absence of complex multi-speakers setups.

We hope this will serve as a model and will encourage more clinicians to assess patient outcomes with technologies like CROS. Along the same lines of clinically accessible methodology, we successfully and effectively used a simple 5-point visual analog scale to acutely measure differences in speech clarity when listening without and with CROS in the head-shadow condition. While the subjects were not blind to CROS use, we believe that this subjective outcome is a true effect and not biased as it was only observed in the head-shadow condition and was aligned with speech recognition outcomes.

The benefit of CROS can be highly dependent on the listening situation in which it is used. CROS use is most beneficial when speech is present on the CROS side. However, speech perception can be degraded when speech is located towards the CI and noise is routed via CROS^(12-17)^. In such instances, the use of mute functionality on the CROS device should be encouraged. One consistent observation across the works of Snapp et al.^(10)^, Dorman et al.^(12)^, Dwyer et al.^(13)^, and Kurien et al.^(20)^ is that while listening in the CI+CROS condition, speech recognition scores become similar irrespective of the location of speech and noise. This means that when using a CROS device in the real world, patients have to be less concerned with how to position themselves to get the best SNR and have a greater awareness of the sounds around them. With increased CROS experience, patients can also adapt their current listening strategies or acquire new ones. Overall, patients report reduced listening effort, increased satisfaction and quality of life with CROS use^(13-15,25,26)^.

One concern can be the retention of the CROS device on the ear. The CROS is a small and light device and patients can worry about losing it. However, use of custom slimtip can provide secure coupling and retention with the ear canal. Finally, there is no low battery indicator but the patients can be taught to test if their CROS is functioning by simply rubbing the CROS microphone gently. When this is done, they hear a scratching-like sound on the CI side confirming that the CROS is active. Patients can also replace their CROS battery every 5^th^ day or so to be certain that their CROS doesn’t lose power during the course of the day.

In terms of limitations of the study, considering that the CROS systems were on loan and the recipients used them only at the time of the study evaluation, it was not possible to establish an acclimatization period.

## CONCLUSION

The present study showed that use of the CROS system helps unilateral CI recipients overcome head-shadow and and achieve better speech understanding in noise, increased speech clarity and increased ease of listening. While the outcomes with bilateral CIs can still be better, especially with regards to localization, access to sounds at the non-implanted ear via CROS can benefit unilateral CI users who cannot use a contralateral hearing aid or undergo bilateral implantation.
